# Transcriptome dataset of ethylene-treated Klutuk Wulung banana

**DOI:** 10.1016/j.dib.2021.107376

**Published:** 2021-09-16

**Authors:** Fenny Martha Dwivany, Husna Nugrahapraja, Lutfi Dewi Nirmala Sari, Rika Rahma Putri, Cindy Novianti

**Affiliations:** a School of Life Sciences and Technology, Institut Teknologi Bandung, Bandung 40132, Indonesia; b Biosciences and Biotechnology Research Center, Institut Teknologi Bandung, Bandung 40132, Indonesia; c Bali International Research Center for Banana, Badung, Bali 80361, Indonesia; d The Research Foundation of Indonesia Biogeography and Biodiversity (INABIG), Bandung, West Java 40115, Indonesia

**Keywords:** Banana, B genome, Ethylene, Klutuk Wulung, Ripening, Transcriptomics

## Abstract

Klutuk Wulung banana (*Musa balbisiana* Colla, BB Group) is a climacteric fruit whose ripening is influenced by ethylene production. This banana fruit has a relatively slow ripening process time and long shelf-life compared with A genome banana (*Musa acuminata*, AA). Bananas are usually harvested at a pre-climacteric stage and ripened artificially by exogenous ethylene. Hence, the application of exogenous ethylene at the pre-climacteric stage can accelerate the Klutuk Wulung banana ripening. However, there is no report regarding the effect of exogenous ethylene treatment on Klutuk Wulung banana global gene expression. The knowledge of global gene expression of ethylene treated Klutuk Wulung banana will help to understand this fruit ripening process. In this study, global gene expression data of untreated and ethylene treated Klutuk Wulung banana fruit during ripening were available. Total RNA was extracted from fruit pulp for differential expressed gene analysis using RNA-Seq. The RNA-Seq results obtained were ranged from 34,565,252 to 44,752,129 total reads, with 80.5% to 86.7% of reads were mapped against Klutuk Wulung banana genome reference derived from The Banana Genome Hub. In total, 29,968,128 to 37,776,907 transcripts were detected. The transcriptome data discussed in this article were deposited into NCBI's Gene Expression Omnibus (GEO) Series with an accession number GSE162077. These data can be used as information to identify gene candidates involved in fruit ripening for the application in banana postharvest program.

**Specification Table**

**Table:** 

Subject Area	Biological Sciences
Specific subject area	Fruit Ripening
Type of data	Transcriptomics data (abundance measurements derived from the data of RNA-seq)
How data were acquired	Illumina HiSeq. 2500 platform
Data format	Raw: fastq.gz files Processed Data: Tab-delimited text files with FPKM values
Parameters for data collection	Transcriptomics of Klutuk Wulung banana pulp of control (untreated) and ethylene-treated conditions
Description of data collection	Total RNA was extracted from pulp of control (untreated) and ethylene-treated Klutuk Wulung banana fruit, then was sequenced using Illumina Hiseq. 2500
Data source location	Bandung, West Java, Indonesia (6°53′28.9"S 107°36′38.3"E)
Data accessibility	NCBI's Gene Expression Omnibus (GEO) with GEO series accession number GSE162077 https://www.ncbi.nlm.nih.gov/geo/query/acc.cgi?acc=GSE162077 Raw data are available at NCBI's Sequence Read Archive (SRA) database (accession number SRP293766) https://www.ncbi.nlm.nih.gov/sra?term=SRP293766

**Value of the Data**

• *Musa balbisiana* Colla is a B genome banana that has a slower ripening process and longer shelf-life than A genome banana. This data provides a potential genetic source for postharvest management.
• This data provides a comprehensive transcriptomic analysis using pair-end sequencing with two to three biological replicate datasets to understand the metabolic pathways affected by ripening and ethylene treatment.
• This data will help to explain the mechanisms of Klutuk Wulung banana fruit ripening and identify the genes that are expressed differently on ethylene treatment.

## 1 Data Description

Klutuk Wulung Banana (*Musa balbisiana* Colla, BB Group) is a climacteric fruit whose ripening is influenced by ethylene. The fruit has a relatively slower ripening process and longer shelf-life than A genome banana (*Musa acuminata*, AA Group). According to Maduwanthi and Marapana [1], bananas are usually harvested at the pre-climacteric stage and ripened artificially by exposure to exogenous ethylene. Plantain bananas (B-content genome) can only be consumed 18 days after harvest without being treated with ethylene at a storage temperature of 25–27 °C, while A genome banana can be consumed 6 days after harvest [2], [3], [4]–5]. Delays of finger-drop or release of fingers and hands from bunches due to maturity in B-content banana were also found in a study conducted by Imsabai et al. [6]. As a result, B genome bananas became the target of postharvest technology development programs with the characteristics of high-stress resistance and long ripening time-related traits [7].

Hence, the application of exogenous ethylene treatment can accelerate Klutuk Wulung banana fruit ripening and the biological process can be revealed by using transcriptomic analysis. In the previous study, the transcriptomic approach was successfully provided the transcriptome data and revealed global gene expression of chitosan-coated and uncoated banana fruit during ripening, hence provided data for identifying candidate genes involved in the delay of fruit ripening by chitosan coating [8]. Therefore, the transcriptomics data of ethylene-treated Klutuk Wulung banana is important to provide basic information for further candidate genes analysis involved in fruit ripening in response to ethylene treatment of B genome banana to design a better postharvest technology and management on bananas.

The data on this article were included the transcriptomics of ethylene-treated (100 µl/L) and untreated (control) of Klutuk Wulung banana (B genome banana). The global gene expression changes from the transcriptomics during ripening of ethylene-treated and untreated Klutuk Wulung bananas were evaluated. The files of transcriptomics dataset, which were generated from 17 libraries of raw data and 10 sets of processed data, has been submitted to Gene Expression Omnibus (GEO) NCBI database [9].

Control and ethylene-treated Klutuk Wulung banana (*M. balbisiana* Colla, BB Group) RNA library were successfully sequenced and the raw data were deposited in NCBI's Sequence Read Archive (SRA) database with an accession number SRP293766 (https://www.ncbi.nlm.nih.gov/sra?term=SRP293766). The raw data statistics could be seen in Table 1. According to Table 1, total nucleotide, total reads, and GC percentage in this study are sufficient for further analysis since the values of total nucleotides and total reads are constant in each replicate. Moreover, good quality of RNA-Seq data using Illumina sequencing has a criterion of about 40– 50% GC content [6]. This GC percentage is almost similar in transcriptomic data analysis of 12 different tissues of *M. acuminata* and *M. balbisiana*, which ranged from 40 to 48% [10]. Moreover, RNA-seq analysis conducted by Dwivany et al. [8] found that the raw data statistics of control and chitosan-coated *M. acuminata* subgroup Cavendish fruit pulp has GC content of 48–51%. This suggests that these data could be used for further analysis, which continued to the transcriptome assembly and data analysis using Tophat2 [12,13] and Cufflinks [13,14] to get the processed data.

**Table 1:** The transcriptome's raw data output statistics of *Musa balbisiana* (BB Group) ‘Klutuk Wulung’ fruit. This data was generated from day 1 control (**K1**), day 1 ethylene-treatment (**E1**), day 7 control (**K7**), and day 7 ethylene-treatment (**E7**) of *Musa balbisiana* (BB Group) ‘Klutuk Wulung’ fruit in each replicate (replicate **A, B**, and **C**) of the paired-end experiment: forward reads (**1**) and reverse reads (**2**).

No.	Samples ID	Total Reads	Total Nucleotides	GC Percentage
1.	KLU_K1_A_2	12,375,633,236	40,978,918	48
2.	KLU_K1_B_1	13,550,373,976	44,752,129	46
3.	KLU_ K1_B_2	13,550,373,976	44,752,129	46
4.	KLU_K7_A_1	12,061,027,152	39,937,176	46
5.	KLU_K7_B_1	11,725,286,504	38,717,505	46
6.	KLU_K7_B_2	11,725,286,504	38,717,506	46
7.	KLU_K7_C_1	13,233,109,084	43,705,956	47
8.	KLU_K7_C_2	13,233,109,084	43,705,957	47
9.	KLU_E1_A_1	10,466,471,984	34,565,252	47
10.	KLU_E1_A_2	10,466,471,984	34,565,252	47
11.	KLU_E1_C_1	13,041,604,240	43,072,452	47
12.	KLU_E1_C_2	13,041,604,240	43,072,452	47
13.	KLU_E7_A_1	11,575,494,202	38,329,451	47
14.	KLU_E7_B_1	13,523,350,412	44,641,478	47
15.	KLU_E7_B_2	13,523,350,412	44,641,479	47
16.	KLU_E7_C_1	11,731,797,926	38,752,036	48
17.	KLU_E7_C_2	11,731,797,926	38,752,037	48

*All RNA-seq raw data can be accessed at NCBI's Sequence Read Archive (SRA) database (accession number: SRP293766).

The statistics summary of processed data is shown as dataset statistics of the mapping result and transcripts detection (Table 2). These data were available at NCBI's Gene Expression Omnibus (GEO) with accession number GSE162077 (https://www.ncbi.nlm.nih.gov/geo/query/acc.cgi?acc=GSE162077). In total, 29,968,128–37,776,907 transcripts were detected with 80.5– 86.7% mapped reads, which indicates a good mapping result. According to Trapnell et al. [13] a good mapping result has a minimum mapped value of 70% to genome reference. Venkataramana et al. [11] also found that 75.55% *M. balbisiana* transcripts of 12 different tissues were mapped against the whole genome reference which could be used for further analysis. Moreover, Dwivany et al. [8] also found that the transcripts of Cavendish banana (*M. acuminata*) fruit pulp were 75.8– 83.8% mapped against the genome reference. Hopefully, this data could be used as a basic information to further analysis of gene candidates involved in fruit ripening using RNA-Seq data, especially on B genome bananas.

**Table 2:** Result of mapping and transcripts detection of *Musa balbisiana* (BB Group) ‘Klutuk Wulung’ fruit transcriptome**.** This data was generated from day 1 control (**K1**), day 1 ethylene-treatment (**E1**), day 7 control (**K7**), and day 7 ethylene-treatment (**E7**) of *Musa balbisiana* (BB Group) ‘Klutuk Wulung’ fruit.

No.	Sample	Mapped Reads (%)	Transcripts Detected	Accession Number
1.	KLU_K1_A*	80.5	32,937,172	GSM4932720
2.	KLU_K1_B	84.4	37,776,907	GSM4932721
3.	KLU_K7_A*	81.1	32,377,792	GSM4932722
4.	KLU_K7_B	84.3	32,627,611	GSM4932723
5.	KLU_K7_C	85.4	37,339,078	GSM4932724
6.	KLU_E1_A	86.7	29,968,128	GSM4932725
7.	KLU_E1_C	86.3	37,161,983	GSM4932726
8.	KLU_E7_A*	81.6	31,245,382	GSM4932727
9.	KLU_E7_B	84.7	37,815,595	GSM4932728
10.	KLU_E7_C	86.5	33,533,434	GSM4932729

⁎ Single-end experiment.

## 2 Experimental Design, Materials and Methods

### 2.1 Plant materials

Klutuk Wulung banana (*M. balbisiana* Colla, BB Group) was harvested from Dago, Bandung, West Java, Indonesia (6°53’28.9”S, 107°36’38.3” E). The criteria for Klutuk Wulung banana used in this study were the relative similarity of fruit skin color, physiological age, and fruit size, and also the absence of fungal infection or physical defects as described by Lustriane et al. [5] regarding the sorting of bananas for research purposes. The fruit then was randomly divided into two groups, i.e. a control group (without ethylene treatment; encoded with *K*) and an ethylene treatment group (encoded with *E*). For the ethylene treatment group, 100 µl/L exogenous ethylene gas was exposed to bananas and carried out in a glass container [15]. Then, the bananas were incubated for 24 h and stored outside the container at room temperature (26 °C ± 1 °C) for 7 days.

### 2.2 Total RNA isolation, library preparation, and sequencing

Total RNA of Klutuk Wulung banana was extracted from banana pulp on the first day (duplicates) and seventh day (triplicates) of fruit ripening using Cordeiro et al.’s method [16]. The RNA concentration was measured using NanoDrop spectrophotometer (Eppendorf BioSpetometer® Kinetic) at the wavelength of 230, 260, and 280, then performed rRNA bands check using electrophoresis technique on 1.5% agarose gel. RNA was then purified from DNA contaminant using DNAseI kit from Thermo Scientific (Catalog Number: EN0521). The RNA library from Klutuk Wulung was constructed using TrueSeq RNA Sample Prep KIT v2 and was sequenced using Illumina platform HiSeq 2500.

Quality control of each sample was managed to examine

### 2.3 Transcriptome assembly and data analysis

Raw sequence quality of each samples were checked using FastQC V0.11.8 program in order to get clean reads from possible Illumina adapters, low base score sequences, and PCR contaminations [10]. The adapter sequence was removed from the raw data by using Trimmomatic V0.38.0 program [17]. Mapping of clean reads were conducted using the Tophat2 V2.1.0 program [12,13] with genome reference *Musa balbisiana* DH PKW V1.1 from The Banana Genome Hub (https://banana-genome-hub.southgreen.fr/organism/Musa/balbisiana) [18]. The mapped reads aligned to the annotated loci, then were normalized and quantified into FPKM value using Cufflinks V2.2.1 [13,14].

## Credit Author Statement

**Fenny Martha Dwivany:** Project administration, Conceptualization, Methodology, Validation, Writing – original draft; **Husna Nugrahapraja:** Resources, Data Curation, Validation, Methodology; **Lutfi Dewi Nurmala Sari:** Formal Analysis, Conducted the experiment; **Rika Rahma Putri:** Formal Analysis, Conducted the experiment, Writing - original draft; **Cindy Novianti:** Writing – review & editing.

## Declaration of Competing Interest

No conflict of interests declared.
